# Isoimperatorin alleviates fungal keratitis by regulating NF-κB pathway and macrophage immune response

**DOI:** 10.3389/fimmu.2026.1676397

**Published:** 2026-01-27

**Authors:** Shiqi Song, Huirong Xu, Mengzhu Liu, Qiang Xu, Ling Wang, Fenglei Wang, Xiaomei Wan, Xiaoyan Sun, Chengye Che

**Affiliations:** 1 Department of Ophthalmology, The Affiliated Hospital of Qingdao University, Qingdao, China; 2 Institute of Pharmacy, Shandong University of Traditional Chinese Medicine, Jinan, China

**Keywords:** fungal keratitis, immune response, isoimperatorin, network pharmacology, TLR4/MyD88/IKK/NF-κB pathway

## Abstract

**Purpose:**

The purpose of this research study was to investigate the impact of isoimperatorin on Aspergillus fumigatus (A. fumigatus) keratitis and elucidate its underlying mechanisms via the TLR4/MyD88/IKK/NF-κB signaling pathway.

**Materials and methods:**

The targets of the active ingredient isoimperatorin were identified via network pharmacology. Key interactions between isoimperatorin and its predicted targets were validated using molecular docking. *In vitro* RAW264.7 macrophage cells of A. fumigatus keratitis, the effects of isoimperatorin on inflammatory signaling and cell apoptosis were assessed using western blotting, immunofluorescence, and flow cytometry. *In vivo* C57BL/6 mouse models of A. fumigatus keratitis, the effects of isoimperatorin on inflammatory signaling, neutrophil infiltration, macrophage polarization, and cell apoptosis were assessed using quantitative real-time polymerase chain reaction (qRT-PCR), western blotting and immunofluorescence.

**Results:**

Isoimperatorin inhibited TLR4/MyD88 complex formation and IKK/NF-κB phosphorylation, downregulated pro-inflammatory cytokines, reduced neutrophil infiltration, and promoted macrophage polarization from the M1 to the M2 phenotype. Additionally, it inhibited cell apoptosis and alleviated corneal epithelial damage.

**Conclusion:**

This study demonstrates that isoimperatorin exerts a protective effect against A. fumigatus keratitis primarily by modulating the host immune response. The mechanism is mediated through the multi-target inhibition of the TLR4/MyD88/IKK/NF-κB pathway, leading to a coordinated reduction in inflammation and tissue damage. These findings highlight the therapeutic potential of isoimperatorin as a novel immunomodulatory strategy for managing fungal keratitis.

## 1 Introduction

Fungal keratitis (FK), also known as mycotic keratitis, is a common corneal infection that frequently leads to blindness, particularly in developing countries. It predominantly affects young male agricultural workers involved in outdoor activities (1, 2). Epidemiological modelling estimates a global annual incidence of at least 1 million cases, with geospatial analysis indicating a disproportionate burden in Asia and Africa, where 84,143–115,697 cases of vision loss have been documented (3). Generally, Aspergillus and Fusarium species are common in tropical and subtropical regions and Candida species are dominant in temperate areas (4). Patients with FK often experience severe infection and inflammation, resulting in corneal opacity and intense ocular pain (2). In spite of advances in drug discovery, the range of available antifungal agents remains limited, comprising mainly polyenes, triazoles, echinocandins, and pyrimidine analogues, which are used either alone or in combination with systemic therapies (2). No new topical antifungal eye drops have been approved since natamycin was introduced in the 1960s (5). However, the effectiveness of 5% topical natamycin is limited by its poor stromal penetration (6). This limitation is exacerbated by the rising prevalence of drug-resistant Fusarium strains and increased biofilm formation (7).

The pathogenesis of FK involves a complex interplay between host immune response and fungal invasion (8). Following corneal colonization, the innate immune system is rapidly activated, with neutrophils playing a key role in the early inflammatory response (9). These cells eliminate pathogens by releasing reactive oxygen species and elastase, but excessive activation contributes to corneal stromal proteolysis and barrier dysfunction (10). Simultaneously, macrophages undergo dynamic phenotypic changes—initial M1 (pro-inflammatory) polarization enhances inflammation through pro-inflammatory cytokines, while subsequent M2 (anti-inflammatory/repair) polarization facilitates tissue repair via interleukin-10 (IL-10) and transforming growth factor-beta (TGF-β) secretion (11). This imbalance also induces a caspase-dependent apoptotic cascade in corneal epithelial cells (12). Aberrant mitochondrial apoptotic signaling not only exacerbates ulceration but also perpetuates immune activation through the release of damage-associated molecular patterns (DAMPs), establishing a self-sustaining cycle of inflammation and apoptosis (13).

Isoimperatorin is a methanolic extract of the roots of Angelica dahurica, which is a naturally occurring coumarin compound (14). Previous studies have found that isoimperatorin has pharmacological effects of anti-inflammatory, analgesic, anti-tumor and antiviral (14). Jiang et al. found that it can induce cancer cell apoptosis by regulating the MAPK/ERK1/2 signaling pathway (15); Fan et al. found that isoimperatorin reduced oxidative stress in periodontal ligament cells, inhibited the secretion of pro-inflammatory factors in periodontal ligament cells and activated the ERK1/2 and nuclear factor kappa B pathways (16). The NF-κB pathway is a central mediator of innate immune and inflammatory responses, while the MAPK/ERK1/2 pathway is critically involved in cell proliferation and stress responses. Dysregulation of these pathways is implicated in the pathogenesis of various inflammatory diseases. Notably, in the context of fungal infections, NF-κB is often activated upstream by pattern recognition receptors such as Toll-like receptor 4 (TLR4), which initiates a signaling cascade via the adaptor protein MyD88 and the IκB kinase (IKK) complex, constituting the TLR4/MyD88/IKK/NF-κB axis. However, the anti-inflammatory mechanism of isoimperatorin, particularly its role in modulating the TLR4/MyD88/IKK/NF-κB signaling in fungal keratitis, remains poorly understood, and its potential application in FK has not been systematically studied.

Network pharmacology (NP) is a powerful tool for elucidating multi-target mechanisms of natural compounds (17). By constructing a comprehensive “drug component target pathway” network, this study systematically explored the therapeutic mechanism of isoimperatorin against FK. Molecular docking provided molecular-level validation of the predicted interactions, offering structural support for the network pharmacology predictions (18). Thus, this present study was aimed to explore the role of isoimperatorin in the treatment of fungal keratitis, as well as its underlying mechanisms based on NP, using RAW264.7 cells, and C57BL/6 mouse models. The findings demonstrated that isoimperatorin attenuated inflammation by modulating the TLR4/MyD88/IKK/NF-κB pathway, significantly reduced neutrophil infiltration, inhibited apoptosis, and promoted pro-inflammatory M1 macrophages towards anti-inflammatory M2 macrophage polarization, thereby highlighting isoimperatorin as a promising therapeutic candidate for treating FK.

## 2 Materials and methods

### 2.1 Isoimperatorin preparation

Isoimperatorin powder (10 mg; MedChemExpress, Cat. No. HY-N0286, CAS No. 482-45-1, China) was dissolved in dimethyl sulfoxide (DMSO (3.6999 mL); Sigma-Aldrich, USA) to prepare a 10 mM stock solution. After preparation, we filtered the stock solution using a TNL2520 sterilized syringe filter (Xiboshi, China), its membrane was made of nylon, with a diameter of 25 mm and a pore size of 0.20 μm. Before each experiment, isoimperatorin was diluted with either phosphate-buffered saline (PBS) or cell culture solution to reach an experimental concentration of 3.125, 6.25, 12.5, 25, 50 and 100 μM.

### 2.2 Network pharmacological analysis of isoimperatorin

Active compounds and potential targets of isoimperatorin were retrieved from the Swiss Target Prediction database. The keyword “fungal keratitis” was used to search the GeneCards, DisGeNET, and OMIM databases. A Venn diagram displaying overlapping targets for FK and isoimperatorin was created using the Venny 2.1 online tool. These targets were then uploaded to the STRING database to construct a protein-protein interaction (PPI) network. Gene Ontology (GO) and Kyoto Encyclopedia of Genes and Genomes (KEGG) pathway enrichment analyses were performed by the Database for Annotation, Visualization and Integrated Discovery (DAVID), with bubble plots generated based on ascending log P-values.

### 2.3 Preparation of A. fumigatus mycelium

The A. fumigatus strain 3.0772 (China General Microbial Culture Collection, Beijing, China) was cultured in Sabouraud’s liquid medium under shaking conditions (120 rpm) at 37°C for 5–7 days. After waiting for the mycelium to clump, place it in a grinding rod for mechanical grinding. After the hyphae are no longer visible to the naked eye, place all hyphae in sterile centrifuge tubes at 4°C, 5000rpm, and centrifuge for 5 minutes. After centrifugation, perform a washing shaking centrifugation cycle using PBS, repeating the process 3 times. The final suspension was diluted to an experimental concentration of approximately 10^8 mycelial fragments/mL. In order to quantify more accurately, the concentration of the final suspension was assessed by measuring the absorbance at 600 nm using a microplate reader (PerkinElmer, USA) and subtracting the absorbance of the blank (PBS group) at the same wavelength. Results showed that the OD_600_ corresponding to the concentration of 10^8 fungal fragments/mL was consistently 0.85 ± 0.05. Live mycelium was used for establishing murine models of A. fumigatus keratitis, while inactivated mycelium (pre-treated with 75% alcohol for 24 hours) was used in cellular experiments. Compared to live mycelium, inactivated mycelium can better avoid excessive activation of host immunity and control the microenvironment during the experimental process. In addition, safety is also an important consideration, so we chose to use inactivated mycelium instead of live mycelium in cell experiments.

### 2.4 Cytotoxicity assay

Cytotoxicity assay was used to investigate the effect of isoimperatorin on cell viability in RAW264.7 cells. Cell viability was assessed using the Cell Counting Kit-8 (CCK-8; Solarbio, Beijing, China). RAW264.7 cells were inoculated into a 96-well plate at a density of 1 × 10^4 cells/well and incubated for approximately 36 h until they reached 75% confluence. Various concentrations of isoimperatorin (3.125, 6.25, 12.5, 25, 50, and 100 µM) were then added to the culture medium and incubated with the cells for 24 h. Next, CCK-8 reagent (10 µL) was added and incubated for a duration of 1.5 h. The cell survival rate was assessed by measuring the absorbance at 450 nm using a microplate reader (PerkinElmer, USA). Cell viability was calculated relative to the control group, which included a vehicle control (0.25% DMSO without isoimperatorin, which had no significant cytotoxicity) and a blank control (culture medium without cells). Cell viability (%) was calculated relative to the control group using the following equation: Viability (%) = [(As - Ab)/(Ac - Ab)] × 100%, where As, Ac, and Ab represent the absorbance of the sample, control (cells with DMSO), and blank (culture medium without cells), respectively. All experiments were performed with at least biological triplicates with each 3 technical replicates (n ≥ 3).

### 2.5 Cell culture and stimulation with A. fumigatus

RAW264.7 murine macrophages (Cell Bank of the Chinese Academy of Sciences, Shanghai, China) were cultured in Dulbecco’s Modified Eagle Medium (DMEM) supplemented with 10% fetal bovine serum (FBS) and 1% penicillin-streptomycin at 37°C in a 5% CO2 atmosphere. RAW264.7 murine macrophages were seeded in 6-well plates at a density of 5 × 10^5 cells/well and cultured for approximately 24 hours until they reached approximately 75% confluence. Prior to stimulation with inactivated A. fumigatus mycelium for a duration of 16 h, cells were pre-treated with isoimperatorin (25 µM) or an equivalent volume of DMSO (0.25%) as the vehicle control for 2 h. The 25 µM of isoimperatorin was chosen through the dose response experiment and the chosen 0.25% DMSO was also done in the dose dependent experiment to show it did not have cytotoxic effects. Subsequently, the liquid in the culture plate was aspirated under ice bath conditions and washed 3 times with PBS. Prepare RIPA buffer (Beyotime, China), PMSF (Beyotime, China), and phosphatase inhibitor (Beyotime, China) in a ratio of 100:1:1. After adding 100ul of prepared RIPA for 5 minutes, scrape it with cell scraping and transfer into an EP tube. After being placed on ice for 2 hours of cracking, centrifuge at 4°C, 12000rpm for 5 minutes, and transfer the supernatant to another new EP tube, which were collected for western blotting analysis. BCA method is used to determine protein concentration and calculate buffer loading.

### 2.6 Cell scratching assay

Cell scratching assay was used to investigate the effect of isoimperatorin on cell migration in RAW264.7 cells. RAW264.7 murine macrophages were seeded at a density of 5 × 10^5 cells/well in a 6-well plate with 1.5 mL culture medium per well using a 1 mL sterile pipette tip. When cell monolayers reached over 90% confluency, a sterile pipette tip was used to create scratches. Migration was monitored at 0, 24, 48, and 72 hours in 25µM isoimperatorin-treated or DMSO-treated serum free media, and cell migration capacity was quantified. Migration was monitored using an EVOS M5000 fluorescence microscope (Thermo Fisher Scientific, USA) at 10 fold magnification at 0, 24, 48, and 72 hours in 25µM isoimperatorin-treated or DMSO-treated serum free media, and cell migration capacity was quantified. Cell migration capacity was quantified by measuring the scratch wound closure area using ImageJ software (National Institutes of Health, USA). The migration rate was calculated with the following formula: Migration rate (%) = [(A_0_ - A_t_)/A_0_] × 100%, where A_0_ represents the initial scratch area at 0 h, and A_t_ represents the remaining scratch area at time t. For each experimental group, assays were performed in three independent biological replicates (n = 3), with 3 wells per group in each experiment, and 5 random fields per well were analyzed.

### 2.7 Draize eye test

The Draize test (19) was performed to evaluate the ocular safety of isoimperatorin in healthy mice. Healthy C57BL/6 mouse were randomly divided to inject 10 μL of 25 µM isoimperatorin solution in the conjunctival sac of the right eye, while the left eye injected an equal volume of 0.25% DMSO solution as a control. 3 corneas per group, a total of 3 mice. Treatments were administered one time daily. On the 1st, 3rd, and 5th day after treatment, healthy mice without corneal damage were subjected to Corneal fluorescein staining (CFS) using 1% sodium fluorescein and observed under a cobalt blue slit lamp microscope to evaluate whether isoimperatorin would cause any ocular surface damage.

### 2.8 Establishment of FK in C57BL/6 mice

Eight-week-old female C57BL/6 mice were obtained from Jinan Pengyue Laboratory Animal Breeding Co., Ltd. (Shandong, China). All animal procedures followed the guidelines of the Association for Research in Vision and Ophthalmology (ARVO). Mice were randomly assigned to four groups: (a) 0.25% DMSO control group, (b) A. fumigatus + 0.25% DMSO group, (c) 25µM isoimperatorin treatment group, and (d) A. fumigatus + 25µM isoimperatorin treatment group. Mice were anesthetized with intraperitoneal sodium pentobarbital at a dose of 50 mg/kg, and the corneal epithelium was gently abraded with a 30-gauge needle. A 33-gauge Hamilton microliter syringe (Hamilton Corp., Switzerland) was used to inject 2.5 µL of fresh A. fumigatus conidia (5.0 × 10^7/mL) into the corneal stroma. After infection in mice, 10μL of 25μM concentration of isoimperatorin solution was injected subcutaneously into the conjunctiva of mice in groups (c) and (d) once a day. The other groups received 10 μL of DMSO of the same concentration. After modeling, corneal lesion progression was daily observed using a slit lamp microscope, with photographic records captured and documented. The severity of keratitis was subsequently graded on a scale of 0 to 12 points (**Supplementary Table S1**), based on three key parameters: ulcer area, corneal opacity, and ulcer morphology. Notably, the keratitis score represents the sum of these three subscores, providing a composite assessment of disease severity (20). All experiments performed in at least three independent biological replicates.

### 2.9 Hematoxylin-eosin staining

To investigate whether 25µM isoimperatorin affects the neutrophil infiltration in the cornea of infected mice, HE staining was performed on corneal tissue sections of mice on the third day of infection. On Day 3, use scissors and tweezers to remove eyeballs of C57BL/6 mice and carefully place each of them in a container containing OCT embedding agent. Then quickly immerse the container in liquid nitrogen to rapidly freeze the eyeball and the OCT embedding agent. Serial sections (8 µm thickness) were prepared using a Frozen pathology slicer (Leica Microsystems, Germany). Sections were fixed in methanol, air-dried, and stored at -20°C. For H&E staining, sections underwent gradient rehydration (95%→90%→85% ethanol, followed by distilled water), hematoxylin staining, 1% HCl-alcohol differentiation, 1% ammonium hydroxide bluing, eosin Y counterstaining, and gradient dehydration (85%→90%→95% ethanol, then absolute ethanol). After xylene clearing, sections were mounted with neutral balsam and imaged at 400x magnification on an EVOS M5000 microscope (Thermo Fisher Scientific, USA).

### 2.10 mRNA extraction and quantitative real-time polymerase chain reaction

qRT-PCR was used to investigate the effect of isoimperatorin on the macrophage polarization in mice corneas. The mice were sacrificed at 3 days after treatment and full corneas of experimental and control eyes were collected. 6 corneas per group, a total of 3 mice. Use TRIzol reagent (TaKaRa, Japan) to lyse corneal samples collected on the third day in preparation to extract total RNA from the samples. RNA concentration was quantified via NanoDrop 2000 spectrophotometer (Thermo Fisher, USA), with absorbance ratios of A260/A280 (1.8–2.1) and A260/A230 (>1.7) confirming protein/RNA purity and organic solvent removal. The cDNA templates were synthesized by reverse transcription using the RT Master Mix for qPCR (ABclonal, China), followed by RT-qPCR using the SYBR Green Fast qPCR Mix (ABclonal, China). Gene expression was quantified via qRT-PCR using the following primers: TNF-α, NOS2, ARG-1, YM-1 and FIZZ-1. TNF-α and NOS2 are canonical markers of classical M1 activation, driven by TLR4/NF-κB signaling. Their upregulation reflects pro-inflammatory macrophage polarization. While ARG-1, YM-1 and FIZZ-1 are prototypical alternative M2 markers, induced during tissue repair and immunosuppression. β-actin served as the internal control. Nucleotide sequences of mouse primers for real-time RT-PCR (**Supplementary Table S1**). Reactions were performed on a StepOne Plus™ Real-Time PCR System (Applied BiosystemsTM, USA) using the following protocol: Hold Stage: 95°C for 3 min (initial denaturation). Cycling Stage (40 cycles): Denaturation: 95°C for 15 s. Annealing/Extension: 60°C for 1 min. Melting Curve Analysis: 95°C for 15 s→60°C for 1 min→95°C for 15 s. Fold changes were calculated using the ΔΔCt method with the formula: Fold Change = 2−ΔΔCt = 2−[(Cttarget−Ctβ-actin )treated−(Cttarget−Ctβ-actin)control]. Data were analyzed using Excel Software and graphed in GraphPad Prism 9.0.

### 2.11 Western blotting analysis

Western blotting analysis was used to investigate the effect of isoimperatorin on target protein of NP, TLR4/MyD88/IKK/NF-κB pathway, neutrophil infiltration and cell apoptosis. For the preparation of *in vivo* samples, execute the mice as needed and remove their corneas with a blade. Prepare RIPA buffer (Beyotime, China), PMSF (Beyotime, China), and phosphatase inhibitor (Beyotime, China) in a ratio of 100:1:1. Place the removed cornea in 200 μl RIPA, with 6 corneas in a group. Use an organizational homogenizer (Qiagen, USA) to thoroughly grind the cornea, 6–8 times per group, each time lasting 2 minutes. After being lysed on ice for 1 hour, homogenates were centrifuged at 12000 rpm for 5 min at 4°C, and supernatants were collected as protein extracts. For the preparation of *in vitro* samples, prepare RAW264.7 macrophage cells of different groups in advance and aspirate the liquid in the culture bottle on ice and slowly add 100ul RIPA configured in the above ratio. After 5 minutes of lysis, transfer the cell fragments and RIPA to an EP tube. After being placed on ice and cracked for 2 hours, centrifuge at 12000 rpm for 5 min at 4°C, and supernatants were collected as protein extracts. Protein concentration was measured using the Beyotime BCA Protein Assay Kit (Beyotime, China) following the manufacturer’s instructions. Briefly, serial dilutions of BSA standards were prepared to generate a standard curve, and sample absorbance was detected at 562 nm using a microplate reader. Protein loading (30–50 µg per lane) was normalized to the standard curve to ensure equal loading across lanes. Proteins were separated via sodium dodecyl sulfate polyacrylamide gel electrophoresis (SDS-PAGE) on 10% or 12% polyacrylamide gels (depending on the molecular weight of target proteins), transferred to polyvinylidene fluoride (PVDF) membranes (Solarbio, China), which was activated with methanol before transfer. Maintain cooling during the film transfer process, using a constant current of 250mA for 60-90min (depending on the molecular weight of target proteins). Then use QuickBlock™ Blocking Buffer (Beyotime, China) to block for 90 minutes. Wash with 1 x PBST for 5 minutes after blocking, repeat 3 times. Membranes were then incubated overnight at 4°C with primary antibodies and β-actin (1:5000, Elabscience), followed by 1 hour of incubation with secondary antibodies. Protein signals were detected using an electrochemiluminescence (ECL) reagent. Primary antibodies included TLR4 (1:1000 Proteintech), MyD88 (1:5000, Proteintech), IKK (1:2000, Abmart), phosphorylated IKK (p-IKK) (1:2000, Abmart), NF-κB (1:200, Novus), phosphorylated NF-κB (p-NF-κB) (1:200, Wanlei), IL-1β (1:800, R&D Systems), Caspase-3 (1:500, Proteintech), Caspase-8 (1:500, Proteintech), STAT3 (1:1000, CST), phosphorylated STAT3 (p-STAT3) (1:2000, CST), SYK (1:1000, CST), and phosphorylated SYK (p-SYK) (1:1000, CST). Wash with 1 x PBST for 5 minutes after incubation of primary antibody, repeat 3 times. Secondary antibodies, goat anti-rabbit IgG and goat anti-mouse IgG (both 1:10,000, ABclonal), were used. Protein bands were visualized using a Uvitec Alliance Q9 chemiluminescence imaging system (Uvitec, Cambridge, UK). Signals were normalized to β-actin and band intensities were quantified using ImageJ software (Version 1.53g; National Institutes of Health, USA). All western blot experiments performed in at least three independent biological replicates.

### 2.12 Immunofluorescence staining

Immunofluorescence staining was used to investigate the effect of isoimperatorin on target protein of NP, TLR4/MyD88/IKK/NF-κB pathway, neutrophil infiltration and macrophage polarization. Eyeballs were embedded in optical coherence tomography compound (SAKURA, USA) and sections were prepared using the same procedures as those employed for HE staining in earlier stages. Slice and bake in a 37°C incubator for 8 hours. Dilute the serum from the same secondary antibody species 10 times with PBS, add 20mL of 5% bovine serum albumin (BSA; Solarbio, China) to each specimen, and incubate it in a wet box at room temperature for 30 minutes. After suctioning off excess blocking solution, add the primary antibody diluted with blocking solution, including STAT3 (1:150, CST, USA, p-STAT3 (1:150, CST, USA), TLR4 (1:150, Proteintech, China), MyD88 (1:150, Proteintech, China), NIMP-R14 (1:150, Santa Cruz, USA) and F4/80 (1:150, Santa Cruz, USA), add 20mL to each specimen, and place the slices in a wet box at 4°C overnight. Wash the unbound primary antibody from the slides with PBS, drop 20u of fluorescent secondary antibody diluted with PBS onto each specimen, including FITC-conjugated goat anti-rat and goat anti-rabbit antibodies (1:50, Elabscience, China), and incubate the slices in a wet box at room temperature for 1 hour. Nuclei were counterstained with 4′,6-diamidino-2-phenylindole (DAPI; Solarbio, China) at a concentration of 1 μg/mL for 10 minutes, wash away the residual solution with PBS, air dry the slices to a slightly wet state, and add anti fluorescence attenuation sealing agent dropwise to each sample and seal the slices. Images were acquired using an EVOS M5000 fluorescence microscope (Thermo Fisher Scientific, USA) equipped with a digital imaging system.

RAW264.7 macrophage cells were seeded on 15-mm-diameter glass bottom cell culture dishes with 1 × 10^5 cells per well, followed by incubating with different formulations for 6 h. Subsequently, the cells were fixed and blocked, followed by incubation at 4°C with anti-NFκB (1:200, Novus) and anti-p-NF-κB (1:200, Wanlei) overnight at 4°C. On the next day, the cells were washed with PBS and incubated with FITC-conjugated goat anti-rat and goat anti-rabbit antibodies (1:50, Elabscience, China) for one hour at room temperature in the dark, mounted with DAPI solution, and photographed via a high-resolution laser scanning confocal microscope (A1R-SIM, Nikon, Japan).

### 2.13 Flow cytometry

Flow cytometry was used to investigate the effect of isoimperatorin on cell apoptosis. RAW264.7 macrophage cells were flushed with PBS and detached from the six-well plate. The medium was then added, and the cells were gently suspended to create a single-cell suspension. The suspension was subjected to centrifugation at 1000g for five minutes, after which the supernatant was discarded, The cells were resuspended in 1 ml of pre-cooled PBS, and the centrifugation process was repeated at 1000g for another five minutes, with the supernatant discarded again. Finally, the cells were then resuspended in binding buffer to achieve a final cell concentration of 1×10^6 cell/mL. Using the Annexin V-FITC Apoptosis Detection Kit (MeilunBio, China), 10 μL of propidium iodide (Pl) stain and 5 μL of Annexin V-FlTC were added to 100 μL of cell suspension (total number of cells is 1×10^5), followed by a 20-minute incubation in the dark. Experimental controls including untreated RAW264.7 cells (negative control) and RAW264.7 cells with only 10 μL of Pl stain or 5 μL of Annexin V-FlTC added (single positive control). Staining was assessed using the Agilent NovoCyte 2060R flow cytometer (Agilent Technologies, USA) with an FITC signal detector. After the experiment is completed, we analyze it through NovoExpress software.

### 2.14 Statistical analysis

Statistical analysis was performed using GraphPad Prism 9.0 (GraphPad Software, San Diego, CA, USA). The Kruskal–Wallis H test was used for multiple group comparisons because raw data often violated normality assumptions due to biological variability in corneal inflammation. An unpaired two-tailed Student’s t-test was applied to evaluate cell migration, as this assay typically involved comparing two independent groups with normally distributed differences. One-way analysis of variance (ANOVA) was used for analyzing grayscale values for western blotting, where 4 groups needed simultaneous mean comparison while controlling family-wise error rates. A P value < 0.05 was considered statistically significant. Data are expressed as mean ± standard error of the mean (SEM). All experiments were conducted with biological triplicates to ensure reproducibility.

## 3 Results

### 3.1 NP analysis

**Figure 1:**
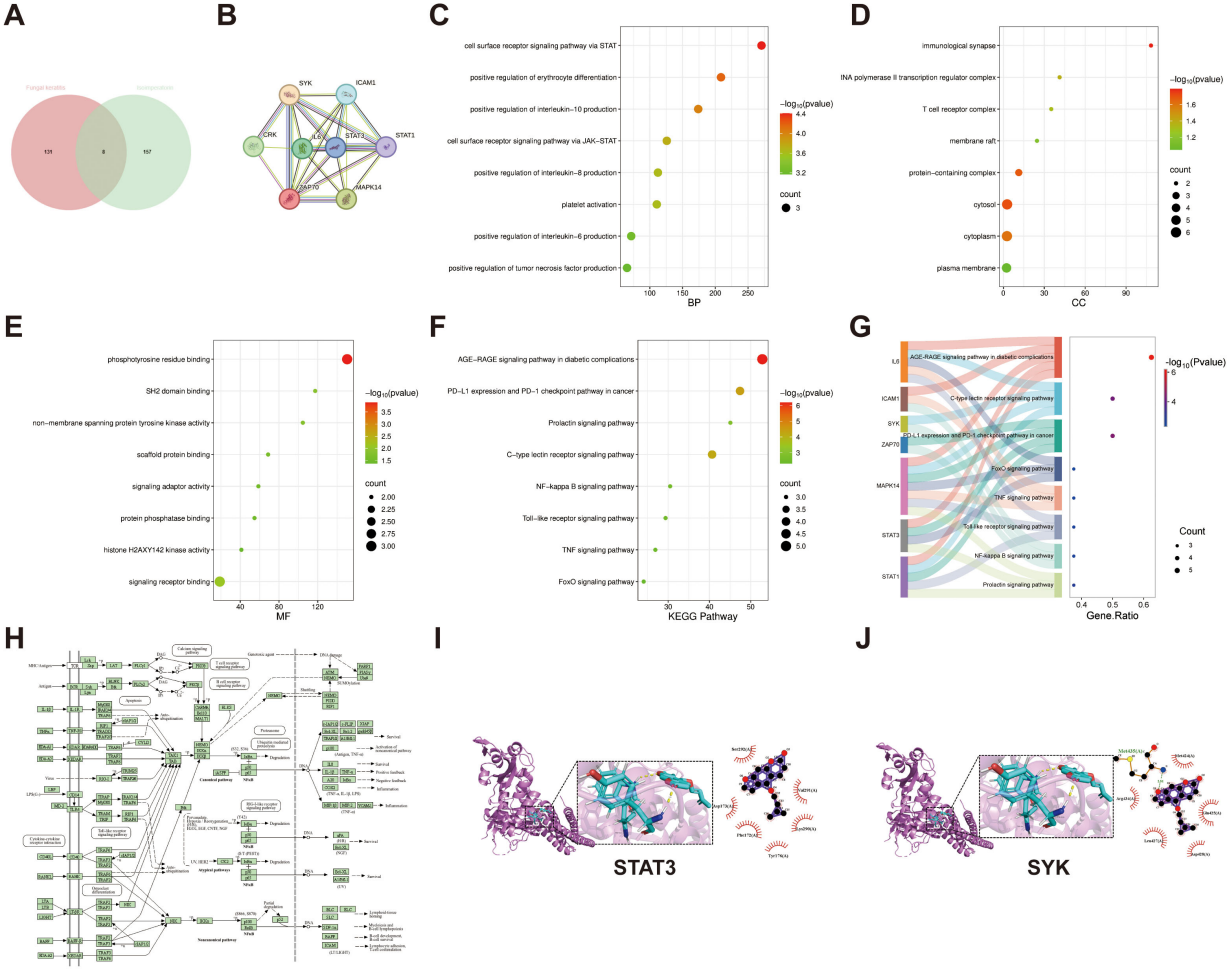
NP analysis of isoimperatorin. **(A)** Venn diagram showing the targets of isoimperatorin and FK. **(B)** The core targets of the PPI network identified using the STRING database. **(C)** Bubble diagram of biological processes (BP). **(D)** Bubble diagram of cellular components (CC). **(E)** Bubble diagram of molecular functions (MF). **(F)** Bubble diagram of the top 8 pathways based on KEGG enrichment analysis. **(G)** Sankey diagram of the KEGG pathway and targets of isoimperatorin in FK treatment. **(H)** The distribution of key targets in the inflammation signaling pathway. **(I)** The binding mode between STAT3 and isoimperatorin in FK. **(J)** The binding mode between SYK and isoimperatorin in FK.

Eight potential drug-disease cross-targets were identified through NP analysis (**Figure 1A**). These targets included STAT1, IL6, SYK, STAT3, ICAM1, ZAP70, CRK and MAPK14 (**Figure 1B**). GO enrichment analysis was performed to explore the molecular mechanisms of isoimperatorin in treating FK (**Figures 1C–E**), where C = BP, D = CC, E = MF. KEGG enrichment analysis showed that the involvement of these 8 genes in NF-κB signaling, Toll-like receptor signaling and TNF signaling pathways (**Figures 1F, G**). Pathways in FK were visualized using R’s pathview package (**Figure 1H**). To further validate the results from NP, molecular docking was used to assess the binding ability. Using AutoDock Vina and PyMol, we simulated the binding of isoimperatorin with potential protein targets. Lower binding energy scores indicated stronger interactions. The top 2 predicted targets were: STAT3 (-3.28 kcal/mol), SYK (-5.61 kcal/mol). Molecular docking visualizations are shown in **Figures 1I, J**, confirming these proteins as potential binding targets. Quantitative results are presented as mean ± SEM from n = 3 independent biological replicates, and experiments were repeated twice to confirm reproducibility.

### 3.2 Effects of isoimperatorin on survival rate, migration of RAW264.7 cells, and toxicity to mouse corneas

**Figure 2:**
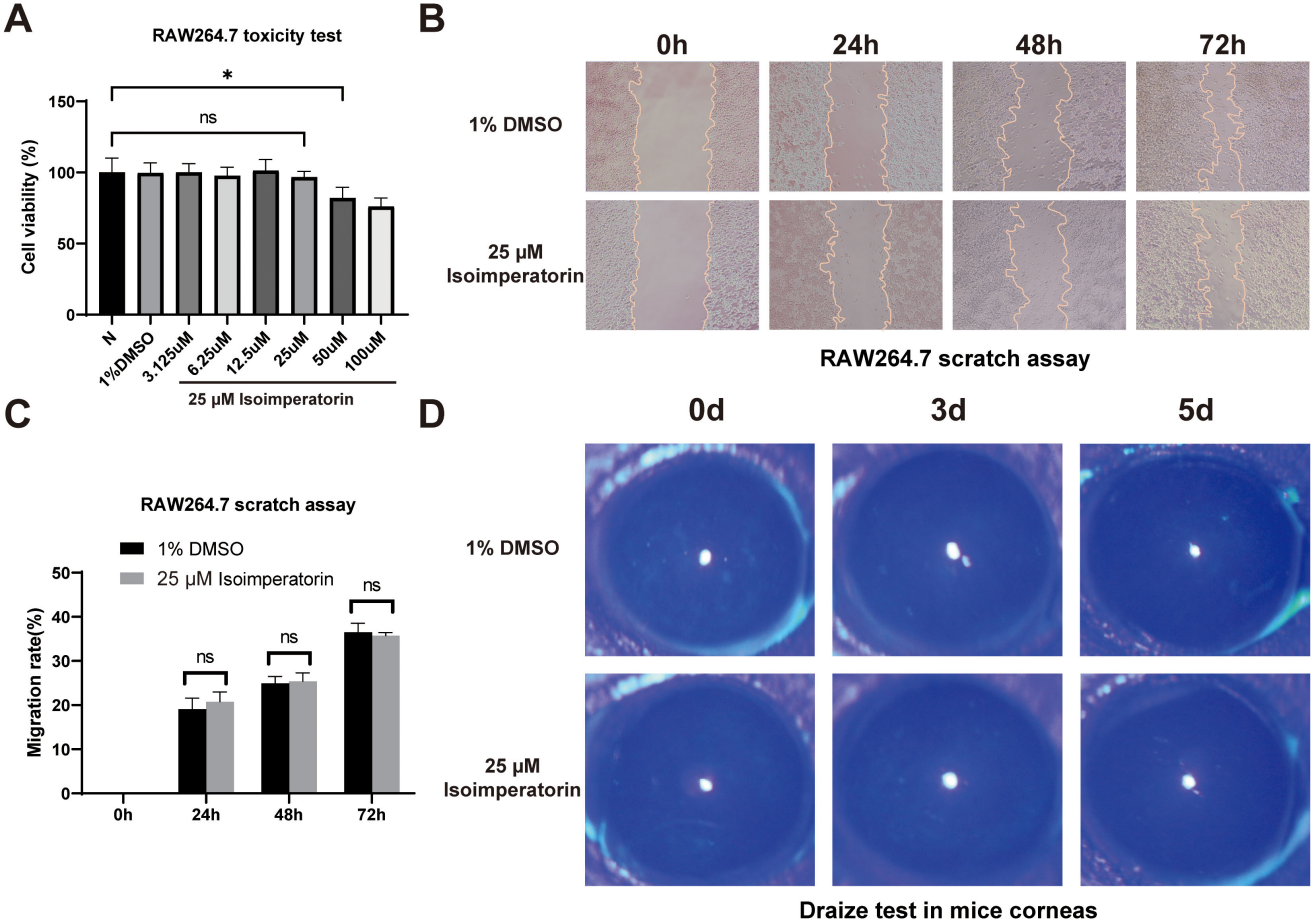
Evaluation of the biocompatibility of isoimperatorin. Cell viability of RAW264.7 cells with isoimperatorin treatment **(A)** Migration area of RAW264.7 cells with isoimperatorin treatment **(B)** and statistical analysis **(C)** Evaluation of isoimperatorin in mouse corneas using the Draize test **(D)** All experiments were performed in triplicate (n = 3), with data presented as mean ± SD and 5 random fields examined per sample. *P<0.05, and ns indicates nonsignificant vs. control group.

CCK-8 assays showed isoimperatorin ranging from 3.125 µM to 25 µM had no effect on RAW264.7 cell viability. In contrast, higher concentrations (50 µM) induced cytotoxicity, and this effect was exacerbated at 100 µM—resulting in severe cell death—as quantified in **Figure 2A**. Cell scratch assays demonstrated no significant differences in migration rates or scratch areas of RAW264.7 cells co-cultured with 25 µM isoimperatorin for 24, 48, and 72 hours compared to the DMSO control group (**Figures 2B, C**). Draize tests performed at 24, 48, and 72 hours post-treatment confirmed no pathological damage to mouse corneas treated with 25 µM isoimperatorin under cobalt blue light (**Figure 2D**). These results indicate that 25 µM isoimperatorin is non-toxic to RAW264.7 cells and mouse corneas.

### 3.3 Isoimperatorin alleviated FK by down-regulating p-STAT3 and p-SYK expression

**Figure 3:**
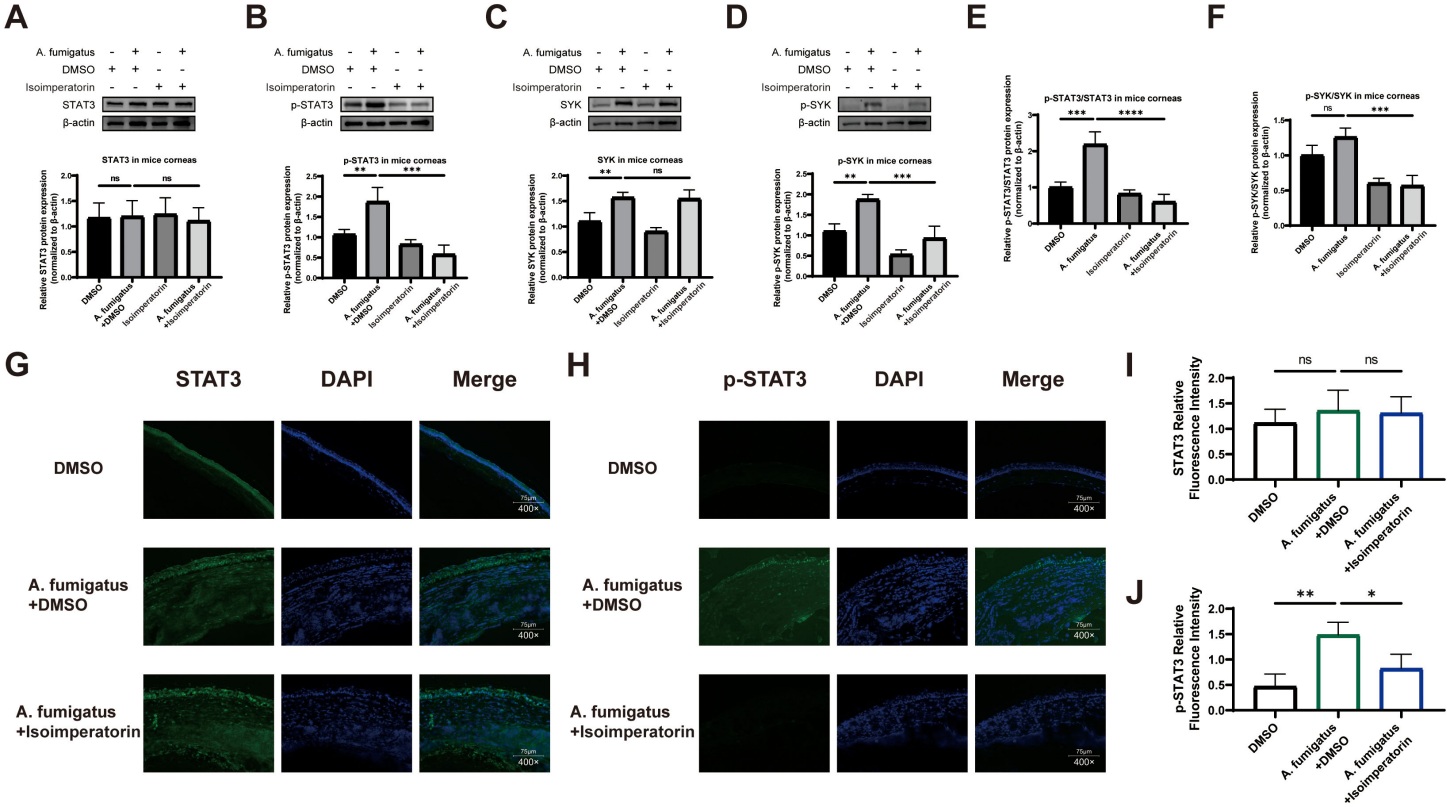
Evaluation of the key targets of isoimperatorin. Changes in protein levels of STAT3 **(A)**, p-STAT3 **(B)**, SYK **(C)**, p-SYK **(D)** p-STAT3/STAT3 **(E)** and p-SYK/SYK **(F)** in mouse corneas. Immunofluorescence staining images of STAT3 **(G)** and p-STAT3 **(H)** in corneas from DMSO, **(A)** fumigatus + DMSO, and **(A)** fumigatus + isoimperatorin groups on day 3 of infection. Statistical analysis of STAT3 **(I)** and p-STAT3 **(J)** relative fluorescence intensity. All experiments were performed in biological triplicate (n = 3), with data presented as mean ± SD and 5 random fields examined per sample. *P<0.05, **P<0.01, ***P<0.001, ****P<0.0001, and ns indicates nonsignificant vs. control group.

Based on NP analysis, STAT3 and SYK were identified as key targets of isoimperatorin in the regulation of FK. **Figures 3A–D** presents representative immunoblots for total STAT3, p-STAT3, total SYK, and p-SYK, respectively, alongside their corresponding quantitative densitometry graphs. Band intensities were quantified using ImageJ software and normalized to β-actin to account for loading variations. Western blot analysis of corneal tissues confirmed that 25 µM isoimperatorin treatment reversed the infection-induced upregulation of p-STAT3 and p-SYK, with β-actin as the loading control to ensure equal protein loading across samples (**Figures 3A–D**). The ratio of phosphorylated STAT3 to STAT3, as well as the ratio of phosphorylated SYK to SYK, further confirms the inhibitory effect of isoimperatorin on the increase in infection induced phosphorylation levels (**Figures 3E, F**). Immunofluorescence staining further supported these findings, showing comparable STAT3 levels in the A. fumigatus + isoimperatorin and A. fumigatus groups (**Figure 3G**), but significantly reduced p-STAT3 staining intensity in A. fumigatus + isoimperatorin group (**Figure 3H**). ImageJ was used to quantify the mean fluorescence intensity (MFI) of STAT3 and p-STAT3 staining. The results demonstrate that there was no significant change in the MFI of STAT3 in the A. fumigatus + isoimperatorin group, while the MFI of p-STAT3 was significantly reduced statistically compared to the A. fumigatus + DMSO group (**Figures 3I, J**). Statistical significance was determined by one-way ANOVA followed by Tukey’s *post-hoc* test, with p < 0.05 considered significant. Together, these results demonstrate that 25 µM isoimperatorin alleviates FK by modulating STAT3 phosphorylation, and SYK activity.

### 3.4 Isoimperatorin-mediated inhibition of TLR4/MyD88/IKK/NF-κB pathway activation *in vivo*

**Figure 4:**
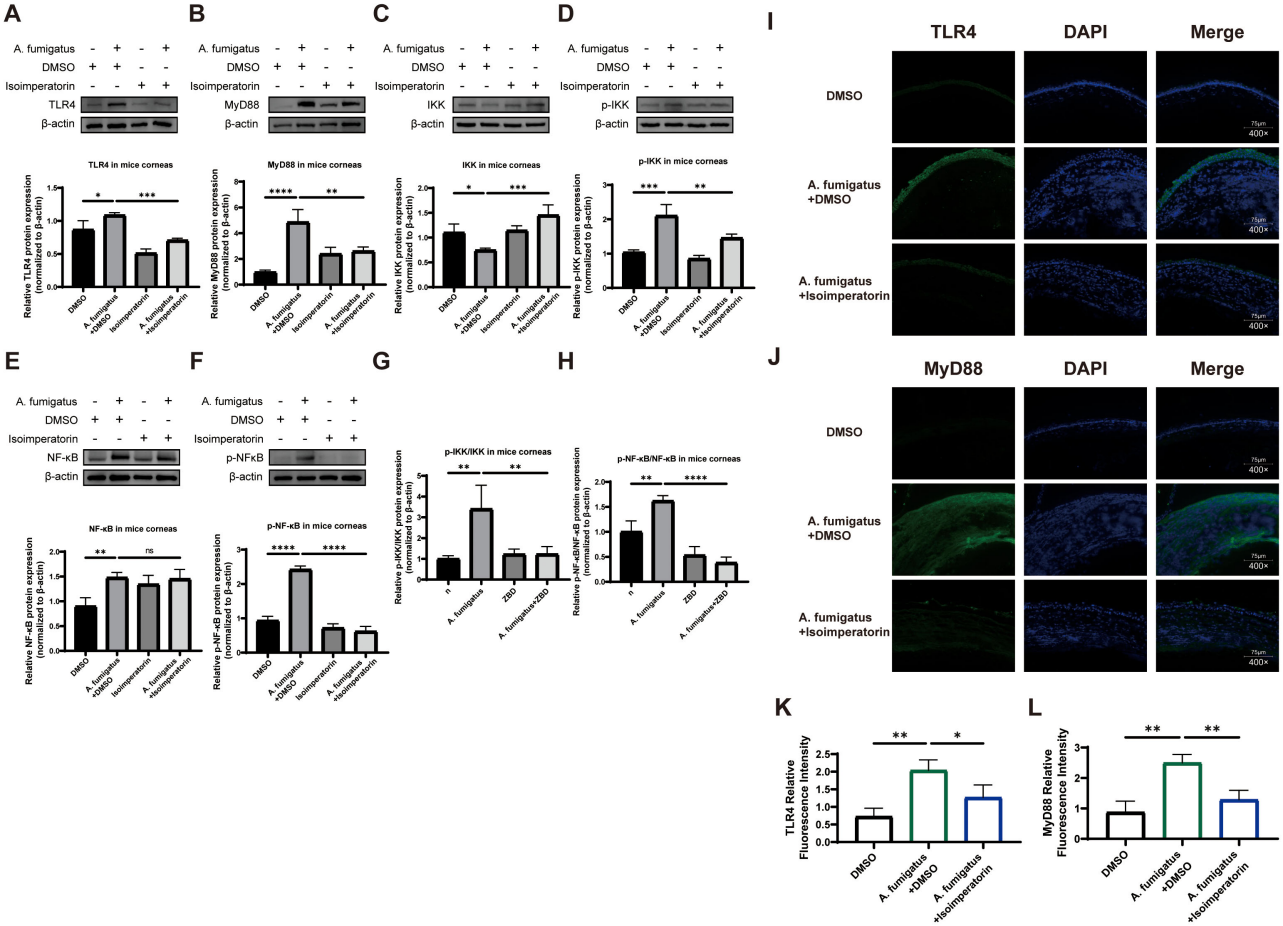
Effect of isoimperatorin on TLR4/MyD88/IKK/NF-κB pathway. Changes in protein levels of TLR4 **(A)**, MyD88 **(B)**, IKK **(C)**, p-IKK **(D)**, NK-κB **(E)**, p-NF-κB **(F)**, p-IKK/IKK **(G)** and p-NF-κB/NF-κB **(H)** in mouse corneas. Immunofluorescence staining images of TLR4 **(I)** and MyD88 **(J)** in corneas from DMSO, **(A)** fumigatus + DMSO and **(A)** fumigatus + isoimperatorin groups on day 3 of infection. Statistical analysis of TLR4 **(K)** and MyD88 **(L)** relative fluorescence intensity. All experiments were performed in triplicate (n = 3), with data presented as mean ± SD and 5 random fields examined per sample. *P<0.05, **P<0.01, ***P<0.001, ****P<0.0001, and ns indicates nonsignificant vs. control group.

**Figure 5:**
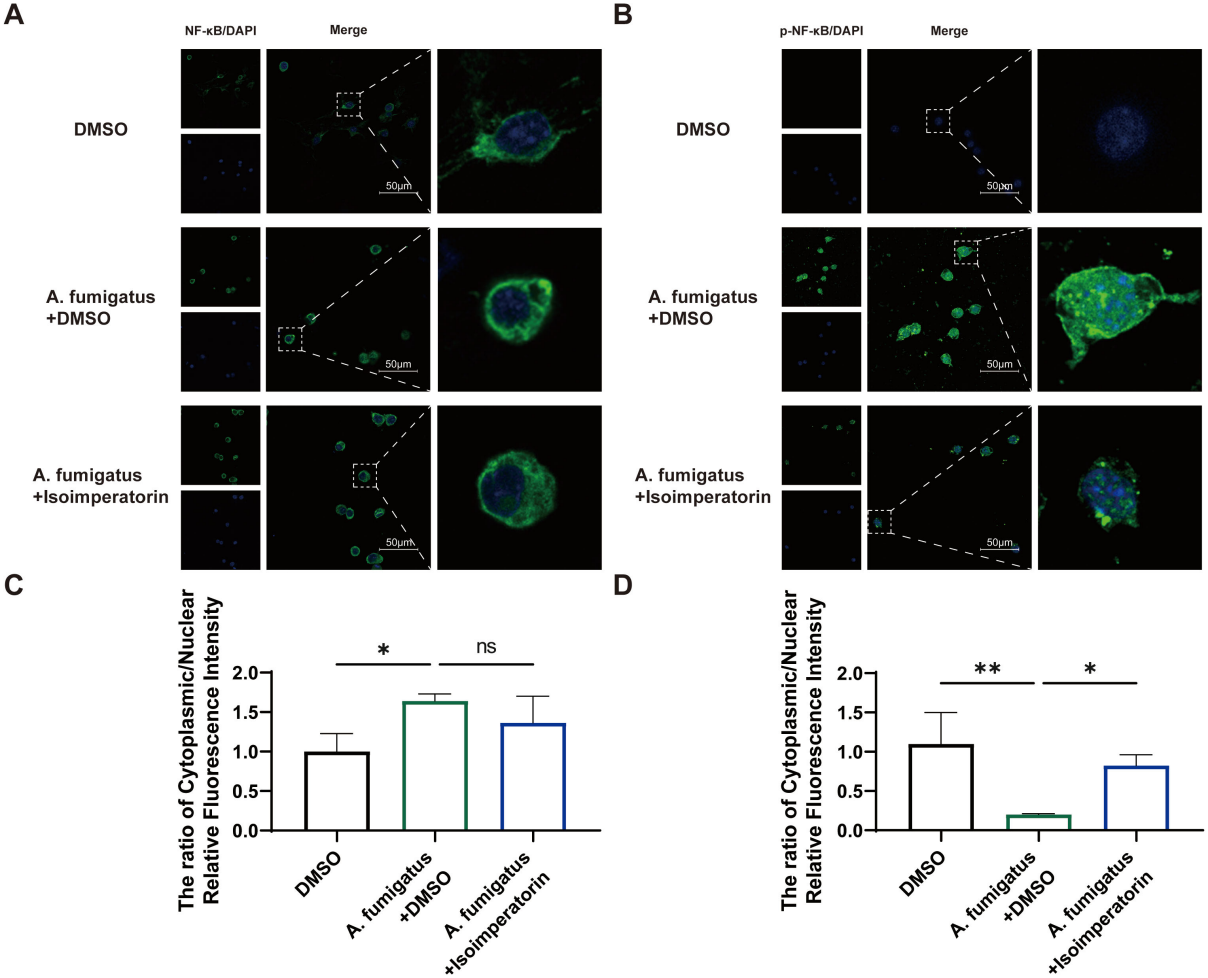
Effect of isoimperatorin on nuclear translocation of NF-κB. Photographs of NF-κB **(A)** and p-NF-κB **(B)** staining on day 3 of infection in the **(A)** fumigatus + DMSO and **(A)** fumigatus + isoimperatorin groups in RAW264.7 cells under confocal microscopy. The ratio of cytoplasmic/nuclear relative fluorescence intensity of NF-κB **(C)** and p-NF-κB **(D)**. All experiments were performed in triplicate (n = 3), with data presented as mean ± SD and 5 random fields examined per sample. *P<0.05, **P<0.01, and ns indicates nonsignificant vs. control group.

KEGG pathway analysis suggests that isoimperatorin may enhance cellular viability through multiple signaling pathways. To explore this mechanism, we evaluated the expression and activation of key target proteins using western blot analysis. As shown in **Figure 4**, A. fumigatus infection significantly upregulated the protein levels of TLR4 (**Figure 4A**) and MyD88 (**Figure 4B**), both of which were suppressed by 25 µM isoimperatorin treatment. β-actin was used as the loading control to ensure equal protein loading across samples. Immunofluorescence staining confirmed these results, showing stronger TLR4 (**Figure 4I**) and MyD88 (**Figure 4J**) signals in the A. fumigatus + DMSO group compared to A. fumigatus + isoimperatorin group. DAPI served as the nuclear counterstain to demarcate cell nuclei and ensure accurate localization of target proteins. ImageJ was used to quantify the MFI of TLR4 and MyD88 staining. The results demonstrate that the MFI of TLR4 and MyD88 were significantly reduced statistically compared to the A. fumigatus + DMSO group (**Figures 4K, L**). Western blot analysis further demonstrated distinct effects on IKK regulation: total IKK levels decreased after infection but were partially restored with 25 µM isoimperatorin treatment (**Figure 4C**), while p-IKK levels increased post-infection and were attenuated by 25 µM isoimperatorin (**Figure 4D**). NF-κB subunits showed differential responses; 25 µM isoimperatorin had no impact on total NF-κB levels (**Figure 4E**), but significantly inhibited p-NF-κB in infected mice (**Figure 4F**). The ratio of phosphorylated IKK to IKK, as well as the ratio of phosphorylated NF-κB to NF-κB, can further confirm that isoimperatori can inhibit the increase in phosphorylation levels induced by infection (**Figures 4G, H**). These findings suggest that 25 µM isoimperatorin selectively inhibits specific phosphorylation-dependent signaling events in the TLR4/MyD88/IKK/NF-κB pathway during fungal infection. Nuclear translocation of NF-κB p65 subunit, a hallmark of pathway activation, was also assessed using high-resolution laser scanning confocal microscope (A1R-SIM, Nikon, Japan) (21). At a magnification of 600 times, we found that after infection with A. fumigatus, the NF-κB p65 subunit in the cytoplasm and p-NF-κB p65 subunit in the nucleus of RAW264.7 cells significantly increased. However, after treatment with 25 µM isoimperatorin, there was no significant change in the NF-κB p65 subunit in the cytoplasm, but the p-NF-κB p65 subunit in the nucleus decreased significantly. Consistent with the western blot analysis in **Figure 4** showing total NF-κB p65 subunit protein levels without significant changes and reduced p-NF-κB p65 subunit protein levels in 25 µM isoimperatorin-treated groups, immunofluorescence staining in **Figure 5** further demonstrated a marked decrease in nuclear p-NF-κB p65 subunit localization in 25 µM isoimperatorin-treated groups, corroborating the suppression of NF-κB p65 subunit activation at both quantitative and spatial levels (**Figures 5A, B**). ImageJ was used to separate the cytoplasmic and nuclear signals for NF-κB p65 and p-NF-κB p65 in the confocal images. The ratio of cytoplasmic and nuclear fluorescence intensity was calculated for cells in each group (**Figures 5C, D**), which shows a significant increase in the cytoplasmic/nuclear ratio of p-NF-κB in the isoimperatorin-treated group, confirming the inhibition of nuclear translocation.

### 3.5 Neutrophil infiltration in mouse corneas inhibited by isoimperatorin

**Figure 6:**
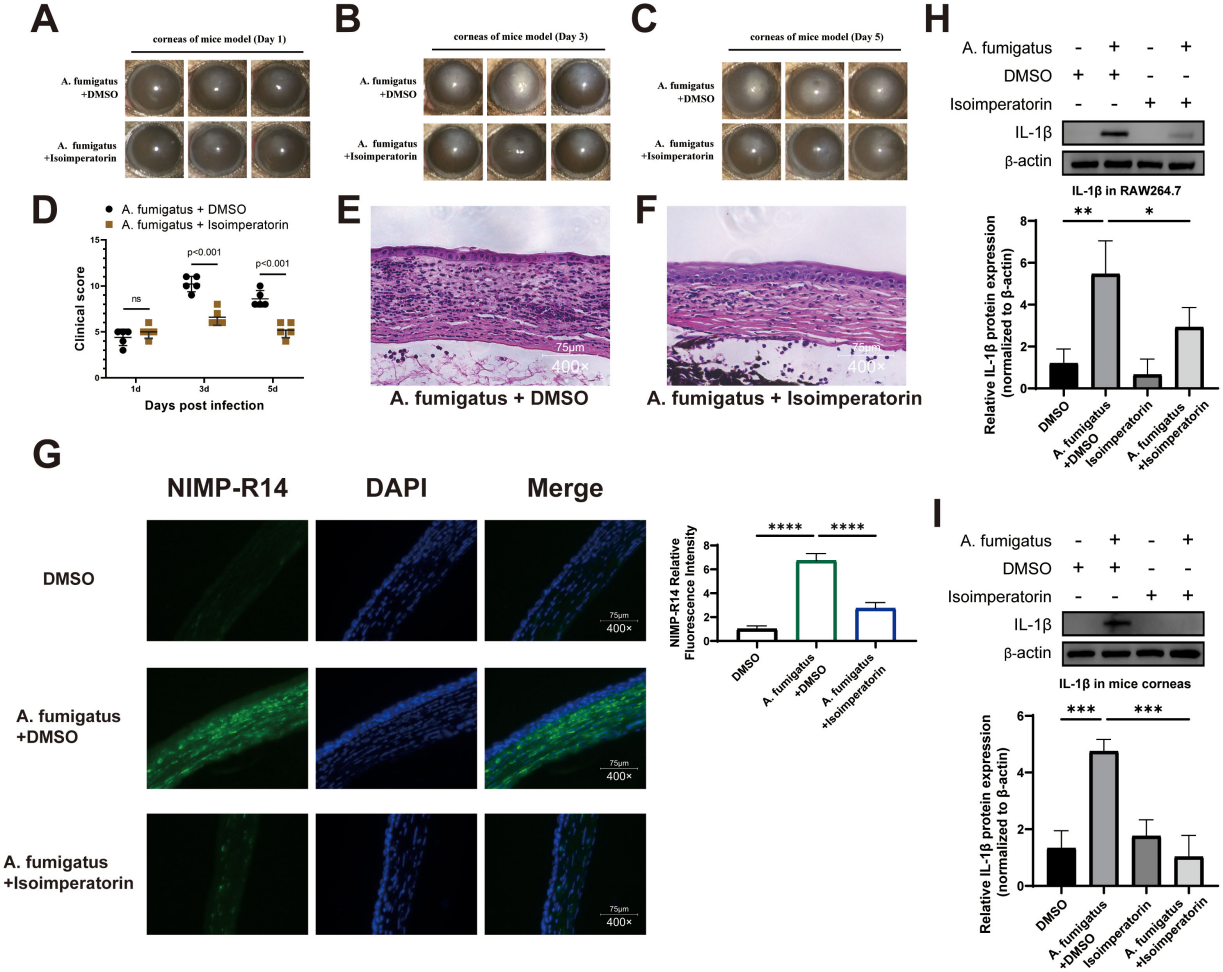
Evaluation of isoimperatorin on neutrophil infiltration in mouse corneas. Representative slit-lamp photographs of **(A)** fumigatus keratitis in mice treated with isoimperatorin or DMSO on days 1 **(A)**, 3 **(B)**, and 5 **(C)**. The clinical scores **(D)** of mice corneas treated with isoimperatorin or DMSO assessed on days 1, 3, and 5 post-infection. Photographs of corneal HE staining on day 3 of infection in the **(A)** fumigatus + DMSO **(E, A)** fumigatus + isoimperatorin **(F)** groups. Immunofluorescence staining images of neutrophils and statistical analysis of NIMP-R14 relative fluorescence intensity **(G)** in corneas from DMSO, **(A)** fumigatus + DMSO and **(A)** fumigatus + isoimperatorin groups on day 3 of infection. Changes in protein levels of IL-1β in RAW264.7 cells **(H)** and mouse corneas **(I)**. All experiments were performed in triplicate (n = 3), with data presented as mean ± SD and 5 random fields examined per sample. P<0.05, *P<0.01, and ns indicates nonsignificant vs. control group.

To evaluate the effect of isoimperatorin on neutrophil infiltration, slit-lamp microscopy was used to examine the condition of the keratitis lesions. Photographs taken on days 1, 3, and 5 revealed reduced severity in the A. fumigatus + isoimperatorin group on days 3 and 5. Photographs (**Figures 6A–C**) showed increased corneal transparency and decreased ulceration in the A. fumigatus + isoimperatorin group. Consistently, clinical scores (**Figure 6D**) showed significantly lower scores in the A. fumigatus + isoimperatorin group compared to the A. fumigatus + DMSO group on days 3 and 5, but were similar on day 1. To assess neutrophil infiltration, corneas from day 3 post-infection were analyzed. HE staining of C57BL/6 mouse eyeball slices (**Figures 6E, F**) showed disrupted epithelial layers and substantial inflammatory neutrophil infiltration in A. fumigatus-treated corneas, while A. fumigatus + isoimperatorin-treated corneas exhibited fewer inflammatory cells, indicating an anti-inflammatory effect. Neutrophil localization in mouse corneas was examined by immunofluorescence using NIMP-R14 (**Figure 6G**). Results showed fewer neutrophils in the A. fumigatus + isoimperatorin group in contrast with the control, consistent with HE staining findings. Inflammatory mediator expression was assessed by measuring corneal tissue lysates and RAW264.7 macrophage cells. Neutrophils are a primary source of IL-1β in inflamed corneas, as evidenced by the concurrent reduction in neutrophil infiltration and IL-1β protein levels in western blotting (**Figures 6H, I**) following 25 µM isoimperatorin treatment. These results confirm that 25 µM isoimperatorin inhibits neutrophil infiltration in both mice and RAW264.7 cells. This correlation reflects the critical role of neutrophil-derived IL-1β in amplifying corneal inflammation, which is dampened by isoimperatorin-mediated inhibition of the TLR4/NF-κB pathway.

### 3.6 Association of isoimperatorin with apoptosis in FK

**Figure 7:**
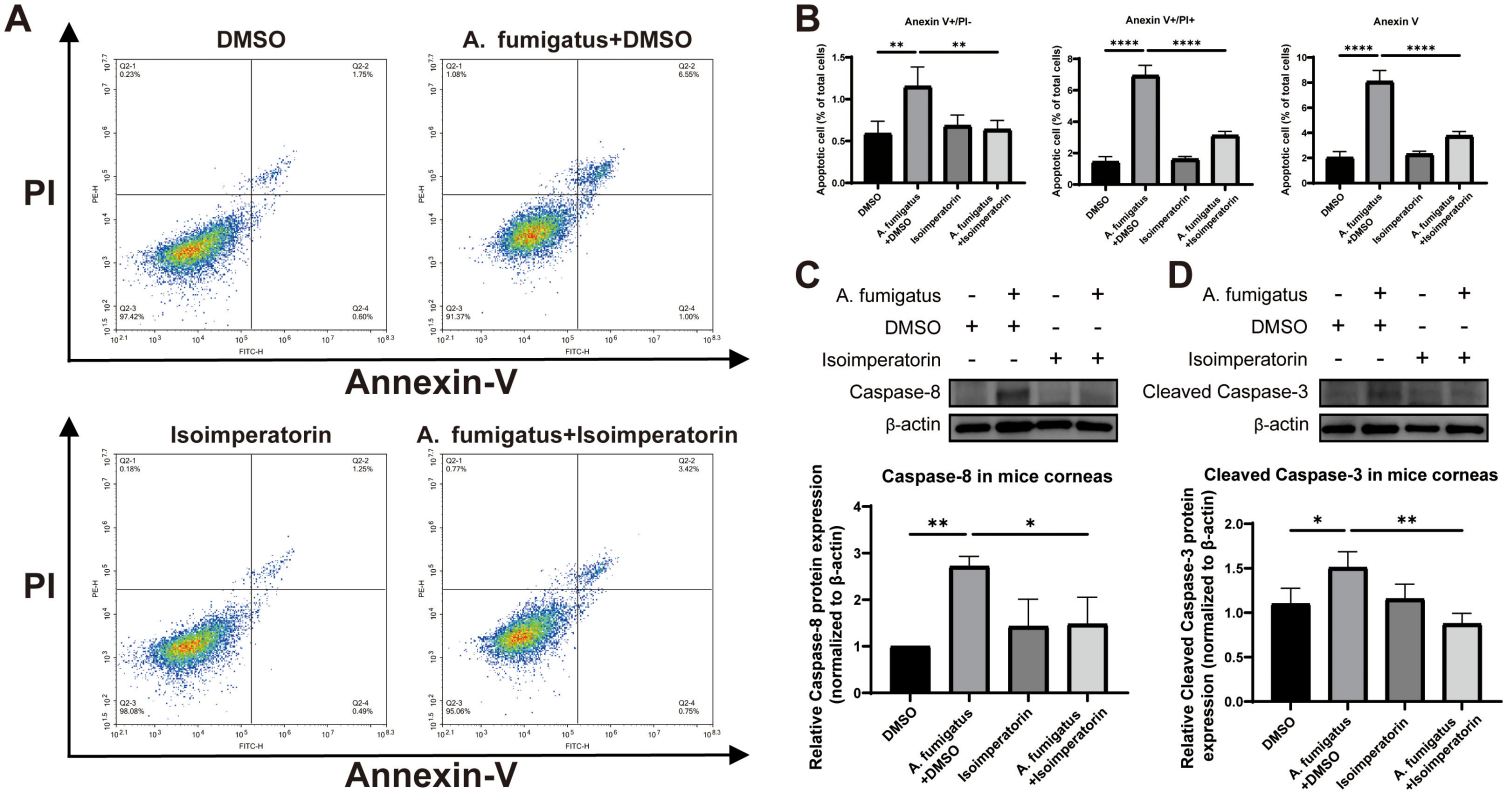
Apoptotic evaluation of isoimperatorin in RAW264.7 cells. Changes in apoptosis of RAW264.7 cells **(A)** and statistical analysis **(B)**. Changes in protein levels of Caspase-8 **(C)** and Cleaved Caspase-3 **(D)** in mouse corneas. All experiments were performed in triplicate (n = 3), with data presented as mean ± SD. *P<0.05, **P<0.01, ****P<0.0001, and ns indicates nonsignificant vs. control group.

To assess apoptosis at the cellular level, Annexin V-APC/7-AAD double-staining flow cytometry was used. Compared with the DMSO control group, A. fumigatus infection increased the proportion of early apoptosis in RAW264.7 cells from 0.587 ± 0.150% to 1.150 ± 0.234%, while treatment with 25µM isoimperatorin significantly reduced the proportion of early apoptosis (0.637 ± 0.110%); Meanwhile, the apoptosis rate of cells in the DMSO treatment group was 1.433 ± 0.344%, while the late apoptosis rate of RAW264.7 cells decreased from 6.927 ± 0.661% to 3.117 ± 0.264% after treatment with 25 µM isoimperatorin. Overall, the total apoptosis rate of cells in the DMSO treatment group was 2.020 ± 0.494%, while the total apoptosis rate decreased from 8.077 ± 0.895% to 3.753 ± 0.362% after treatment with 25 µM isoimperatorin. (**Figures 7A, B**). In addition, studies have shown that Caspase-8 can directly cleave Caspase-3 to induce cell apoptosis (22). Therefore, we further validated the effect of 25µM isoimperatorin on cell apoptosis at the protein level through western blotting analysis. The result shows that infection enhanced Caspase-8 and Cleaved Caspase-3 protein levels in mouse corneas compared to controls and 25 µM isoimperatorin treatment suppressed this infection-induced increase (**Figures 7C, D**).

### 3.7 Effect of isoimperatorin on macrophage recruitment and polarization in mice A. fumigatus keratitis

**Figure 8:**
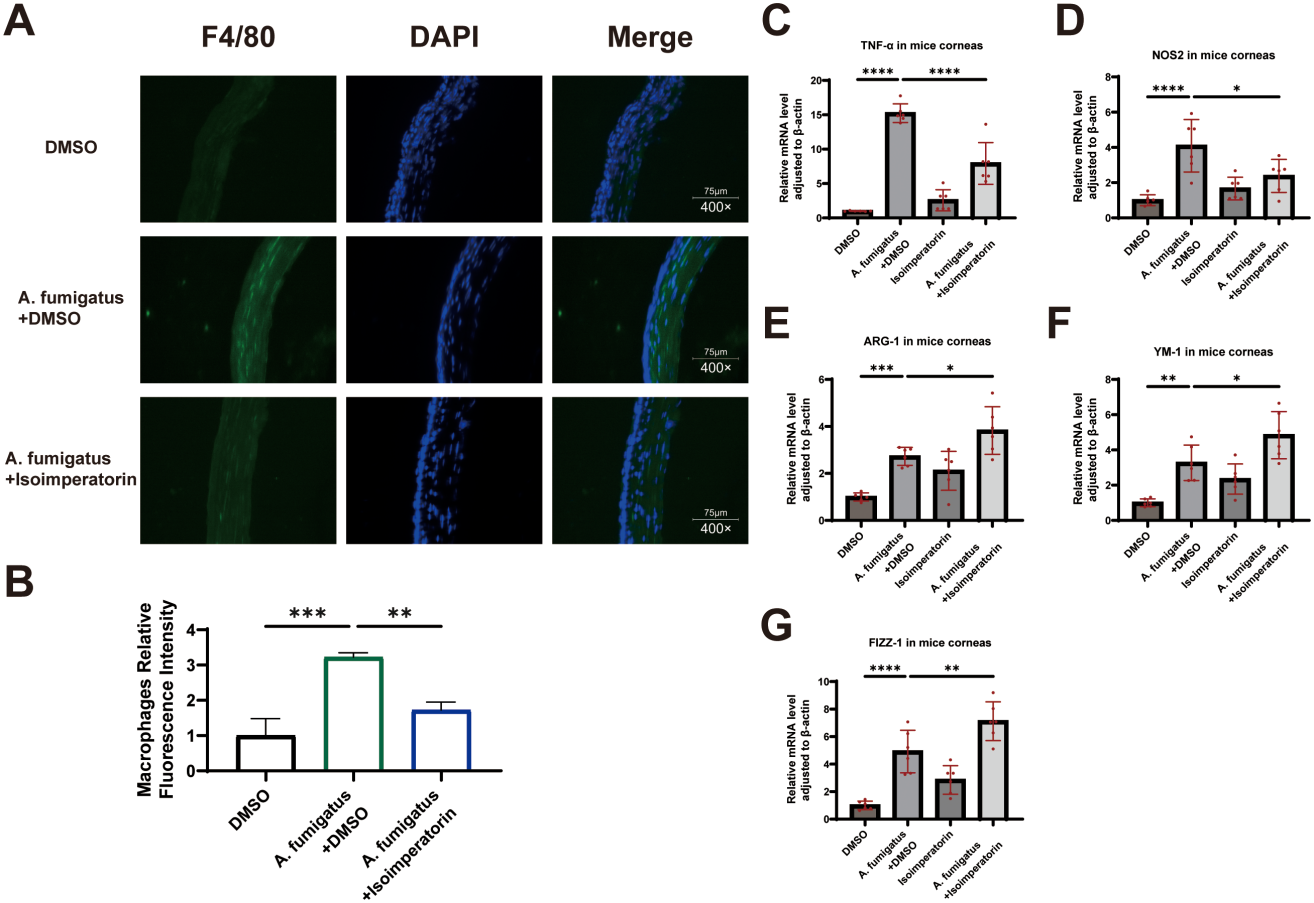
Evaluation of isoimperatorin on macrophages polarization in mouse corneas. Immunofluorescence staining images of F4/80 **(A)** in corneas from DMSO, **(A)** fumigatus + DMSO and **(A)** fumigatus + isoimperatorin groups on day 3 of infection. Statistical analysis of macrophages **(B)** relative fluorescence intensity. The expressions of TNF-α, NOS2, ARG-1, YM-1 and FIZZ-1 were examined by RT-PCR in mouse corneas following **(A)** fumigatus infection at 3 days **(C-G)**. Mouse corneas (6/group). All experiments were performed in triplicate (n = 3), with data presented as mean ± SD and 5 random fields examined per sample. *P<0.05, **P<0.01, ***P<0.001, ****P<0.0001, and ns indicates nonsignificant vs. control group.

Immunofluorescence (**Figure 8A**) revealed a significant increase in macrophage infiltration into the corneal stroma on day 3 post-A. fumigatus infection compared to the normal group. In the A. fumigatus + isoimperatorin group, macrophage numbers were obviously reduced. These results suggest that isoimperatorin inhibits macrophage recruitment to corneal tissue in A. fumigatus-infected mice. ImageJ was used to quantify the MFI of macrophage staining. The results demonstrate that the MFI of macrophage were significantly reduced statistically compared to the A. fumigatus + DMSO group (**Figure 8B**). Compared with the control group, the expression levels of macrophage phenotype-related M1 (TNF-a and NOS2) and M2 (ARG-1, YM-1 and FIZZ-1) cytokine mRNAs increased significantly 3 days after A. fumigatus infected the corneas of mice (P < 0.05). After treatment with isoimperatorin, we found a significant decrease in the mRNA expression levels of TNF-a and NOS2 (**Figures 8C, D**), while the mRNA expression levels of ARG-1, YM-1, and FIZZ-1 increased to varying degrees (**Figures 8E–G**), all of which were statistically significant. These results indicate that A. fumigatus infection can cause the aggregation of macrophages in the cornea of C57BL/6 mice and increase the expression of M1 and M2 cytokines related to macrophage phenotype. Isoimperatorin can inhibit macrophage aggregation caused by A. fumigatus and promote the expression of macrophage phenotype-related M2 cytokines while inhibiting the expression of M1 related cytokines.

### 3.8 The antifungal effect of isoimperatorin

The methods of antifungal experiments have been compiled in the **Supplementary Materials**. To explore the antifungal effect of 25 μM isoimperatorin on A. fumigatus, we conducted a series of experiments, including MIC, biofilm inhibition test and PI staining. The MIC results showed that isoimperatorin had no significant inhibitory effect between 6.25-100 μM (**Supplementary Figure S1**). Biofilm formation is one of the main mechanisms of fungal resistance formation. The production of fungal cell biofilm played a crucial role in fungal resistance to clinical drugs. However, the biofilm inhibition experiment showed that 25 μM isoimperatorin did not exhibit any inhibitory effect on the biofilm of A. fumigatus (**Supplementary Figure S2**). Fungal cell membranes are crucial in maintaining cell homeostasis and protecting cell stability. PI staining indicated that compared with the control group, there was no significant damage observed to the mycelium of A. fumigatus after treatment with 25 μM isoimperatorin (**Supplementary Figure S3**). Corneal homogenates collected on the third day post-infection were cultured on Sabouraud agar medium to assess fungal loads, the corneal homogenates from the DMSO and 25 μM isoimperatorin intervention group cultured a large number of A. fumigatus colonies (**Supplementary Figure S4**). The above experiments demonstrate that there is no clear evidence to suggest that 25 μM isoimperatorin has antifungal activity.

## 4 Discussion

This study ultimately identified 8 drug-disease cross-targets through NP. PPI network analysis revealed that core targets such as STAT3 and SYK played critical roles in anti-inflammatory and immune regulation. The enrichment of GO and KEGG pathways further indicated that the TLR4/MyD88/IKK/NF-κB signaling pathway might be the core regulatory axis. The molecular docking results showed that the active ingredients in isoimperatorin had high affinity for key targets such as SYK and STAT3, verifying the reliability of the NP prediction. The benzopyran ring of isoimperatorin can be inserted into the SH2 domain of STAT3, inhibiting STAT3 dimerization and nuclear translocation through hydrophobic interactions and hydrogen bonding networks (23).

The NF-κB signaling pathway was significantly activated in fungal infections, and 25 µM isoimperatorin selectively blocked the inflammatory cascade by inhibiting the phosphorylation nodes of this pathway (24). This finding was highly consistent with the TLR4/NF-κB targeting predicted by NP, confirming that 25 µM isoimperatorin exerted its anti-inflammatory and immune regulatory effects by acting synergistically on key nodes in the core pathway through multiple components.

Experimental validation showed that the infection of A. fumigatus significantly increased the expression of TLR4 and MyD88 proteins, as well as the phosphorylation level of IKK in corneal tissue, while 25 µM isoimperatorin intervention effectively inhibited these changes. TLR4, as a pattern recognition receptor, recognizes fungal pathogen-associated molecular patterns and activates the IKK complex through the MyD88-dependent pathway, leading to phosphorylation and nuclear translocation of the NF-κB p65 subunit, which further induces the release of inflammatory factors like TNF-α and IL-1β (25). This study is the first to find that 25 µM isoimperatorin may regulate the TLR4/MyD88/IKK/NF-κB pathway through a dual mechanism: on one hand, it directly inhibits the formation of TLR4/MyD88 complexes, reducing downstream signaling. Additionally, by regulating the phosphorylation status of IKK, NF-κB activation is blocked. Although the total NF-κB protein level did not change significantly, its phosphorylation level was significantly reduced, suggesting that 25 µM isoimperatorin selectively inhibits the activation state of NF-κB rather than its expression level. It is worth noting that, despite showing no direct antifungal activity against A. fumigatus *in vitro*, isoimperatorin effectively mitigated corneal inflammation and tissue damage by modulating host immune responses—underscoring its therapeutic effects are primarily mediated through host immunity rather than direct fungal killing. This finding provides a new strategy for antifungal therapy targeting the TLR4/NF-κB pathway.

25 µM isoimperatorin obviously reduced inflammatory cytokines (TNF-α, IL-1β) by regulating the TLR4/NF-κB pathway, while also regulating the expression of chemokines and reducing neutrophil infiltration. HE staining and neutrophil immunofluorescence showed that the corneal neutrophil count within the isoimperatorin-treated group was lower in contrast with the infection group, consistent with the improvement in clinical scores. In addition, 25 µM isoimperatorin reshaped the immune microenvironment by regulating the polarization of M1/M2 macrophages: RT-PCR showed a reduction in the expression of macrophage phenotype-related M1 cytokines (pro-inflammatory) and an increase in the expression of macrophage phenotype-related M2 cytokines (anti-inflammatory), suggesting that 25 µM isoimperatorin may inhibit excessive inflammatory responses by promoting the transformation to an anti-inflammatory phenotype.

The NP and experimental analyses highlighted STAT3 and SYK as key molecules potentially mediating isoimperatorin’s therapeutic effects in fungal keratitis. STAT3, a transcription factor activated by JAKs or cytokine receptors, modulates TLR4/NF-κB signaling through multiple mechanisms (26). First, TLR4 activation induces IL-6 secretion, which binds to gp130/IL-6R on neighboring cells, activating JAK1/STAT3 (27). Nuclear STAT3 then directly binds to promoters of NF-κB target genes, enhancing their transcriptional activity—a feedback loop amplifying inflammation (28). Second, STAT3 physically associates with NF-κB p65 via its SH2 domain, stabilizing NF-κB-DNA binding and prolonging its nuclear retention (29). Third, STAT3 recruits histone acetyltransferases to NF-κB target genes, increasing chromatin accessibility and transcriptional output (30). SYK, a non-receptor tyrosine kinase, acts upstream and in parallel to STAT3. TLR4 engagement recruits SYK via interactions with the TIR domain adapter TIRAP, enhancing MyD88 recruitment and IRAK activation (31). SYK directly phosphorylates TRAF6 on tyrosine residues, accelerating its K63-linked ubiquitination and subsequent IKK activation (32). Additionally, SYK activates PI3K, which phosphorylates AKT; AKT then inhibits GSK-3β, a negative regulator of NF-κB, further stabilizing its activity (33). Together, STAT3 and SYK act as synergistic amplifiers of TLR4/NF-κB signaling: SYK drives immediate TRAF6/IKK activation to initiate NF-κB nuclear translocation, while STAT3, activated by cytokines downstream of NF-κB, sustains activity through transcriptional and epigenetic mechanisms. Its inhibition by isoimperatorin suggesting it may act as a upstream regulator of inflammatory cascades. Future work will delve into the impact of SYK and STAT3 on fungal keratitis to supplement our current observations.

Various experiments demonstrated that 25 µM isoimperatorin reduced corneal tissue damage by inhibiting the Caspase-8/Cleaved Caspase-3 apoptotic pathway. The activation of Cleaved Caspase-3 induced by A. fumigatus infection was significantly inhibited by 25 µM isoimperatorin, indicating that it can maintain tissue homeostasis by regulating the balance of cell apoptosis. While *in vitro* epithelial cell models could further dissect isoimperatorin’s direct effects on epithelial survival, our study prioritizes macrophage-mediated immunomodulation as the primary mechanism driving therapeutic outcomes. *In vivo* data including reduced epithelial apoptosis (cleaved caspase-3/8) and improved histopathology already confirm its protective role in corneal tissue. Interestingly, the anti-apoptotic effect of 25 µM isoimperatorin was also associated with its inhibition of the NF-κB pathway: NF-κB activation could upregulate anti-apoptotic proteins, while 25 µM isoimperatorin indirectly regulated the expression of apoptosis-related proteins by inhibiting NF-κB phosphorylation, thereby protecting corneal epithelial barrier function (34).

Notably, several limitations of this study warrant acknowledgment. First, our preclinical evaluations relied primarily on murine models of A. fumigatus keratitis, which, while valuable, may not fully recapitulate the complexity of human fungal keratitis—including differences in corneal thickness, immune cell composition, and comorbidities such as diabetes that often complicate human disease. Second, the 25 µM concentration of isoimperatorin was selected based on *in vitro* optimization and *in vivo* efficacy, but its pharmacokinetic profile in humans remains uncharacterized, potentially limiting direct translation to clinical dosing. Third, while we focused on macrophage-mediated mechanisms, the role of other immune cells and non-immune cells in isoimperatorin’s action was not exhaustively explored. Fourth, though molecular docking predicted high affinity for SYK/STAT3, biophysical assays to validate these interactions *in vitro* were not performed, leaving room for mechanistic refinement. Finally, this study only used female mice, which may not fully capture sex-specific differences in immune responses or isoimperatorin’s pharmacodynamics. Therefore, future work should investigate potential disparities in macrophage polarization, NF-κB signaling, or drug metabolism between genders. Addressing these limitations in future studies will strengthen the translational potential of our findings and provide a more comprehensive understanding of isoimperatorin’s therapeutic scope.

## 5 Conclusion

Based on NP analysis, our study systematically revealed, for the first time, the multidimensional mechanism underlying the inhibitory effect of 25 µM isoimperatorin on FK, mediated through the TLR4/MyD88/IKK/NF-κB signaling pathway. By inhibiting neutrophil recruitment, reducing cell apoptosis and promoting macrophage polarization from M1 to M2, 25 µM isoimperatorin demonstrated a protective effect against A. fumigatus keratitis.

## Data Availability

The datasets presented in this study can be found in online repositories. The names of the repository/repositories and accession number(s) can be found in the article/**Supplementary Material**.
