# Supplementary material for: Isoimperatorin alleviates fungal keratitis by regulating NF-κB pathway and macrophage immune response
Source: Front Immunol. 2026 Jan 27;17:1676397. doi: 10.3389/fimmu.2026.1676397 (PMC12885997; doi:10.3389/fimmu.2026.1676397)
Supplement: Supplementary file 2 [file DataSheet2.pdf]

Supplementary Material

Supplementary Figures and Tables

1 Supplementary Figures

1.1 Materials and Methods

1.1.1 MIC Experiment

Dilute the spore suspension with Sabouraud medium to 1×10^5 CFU/mL, and then inoculate it into a 96 well plate, followed by the addition of isoimperatorin to achieve final concentrations of 6.25, 12.5, 25, 50 and 100 μM, respectively. The 96-well plate was then incubated in a constant temperature incubator (37 °C, 5% CO2) for 24 h to allow for co-cultivation of the compounds with the spores. The absorbance at 540 nm was measured as soon as the incubation period.

1.1.2 Biofilm Inhibition Experiment

The spore suspension of 1×10^5 CFU/mL and 25μM isoimperatorin were added to the 24-well plate. The spore and isoimperatorin were cultured in a constant temperature incubator (37 °C, 5% CO2) for 24 h until thin hyphae grew. Then, the surface hyphae were removed with an insulin needle, and the Sabouraud medium was extracted. The biofilm in the bottom of the 24-well plate was washed with PBS 3 times and dried at room temperature. Once drying, the 99% methanol was added to the 24-well plate to fix the biofilm for 20 min. After removing the methanol, the biofilm was rinsed with sterile deionized water and dried at room temperature. Crystal violet stain was added to the plate and soaked at room temperature. After 15 min, the dye solution was removed, and the unbound crystal violet was fully washed with sterilized PBS. Then, the biofilm was fully decolorized with 95% ethanol. The mixture of ethanol and crystal violet was absorbed and transferred to a new 96-well plate, and the optical density was measured at 570 nm.

1.1.3 Propidium Iodide (PI)

The spore suspension of 1×10^5 CFU/mL was inoculated with a 6-well plate, and a layer of hyphae was formed later on incubation in a constant temperature incubator (37 °C, 5% CO2) for 24 h. 25μM isoimperatorin was covered on the surface of hyphae. The incubation continued for 24 h, and then the culture solution was sucked out. The hyphae were washed 3 times with sterile PBS and then stained with PI staining solution (Solarbio, Beijing, China). Then, the hyphae were incubated for 15 min and protected from light. The hyphae were photographed with an EVOS M5000 microscope (Thermo Fisher Scientific, America).

1.1.4 Corneal Colony Count

Corneas from mice infected with A. fumigatus for 3 days were collected in pairs. The experimental group was 25 μM isoimperatorin treatment group, and the control group was DMSO treatment group. They were homogenized in 200 μL of sterile PBS using ultrasound and then cultured on Sabouraud agar medium. After 36 hours of incubation (37°C), colony counts of Aspergillus fumigatus were recorded, and photographs were taken.

1.2 Results

To explore the antifungal effect of 25 μM isoimperatorin on A. fumigatus, we conducted a series of experiments, including MIC, biofilm inhibition test and PI staining. The MIC results showed that isoimperatorin had no significant inhibitory effect between 6.25-100 μM (Supplementary Figure 1).

Biofilm formation is one of the main mechanisms of fungal resistance formation. The production of fungal cell biofilm played a crucial role in fungal resistance to clinical drugs. However, the biofilm inhibition experiment showed that 25 μM isoimperatorin did not exhibit any inhibitory effect on the biofilm of A. fumigatus (Supplementary Figure 2).

Fungal cell membranes are crucial in maintaining cell homeostasis and protecting cell stability. PI staining indicated that compared with the control group, there was no significant damage observed to the mycelium of A. fumigatus after treatment with 25 μM isoimperatorin (Supplementary Figure 3).

Corneal homogenates collected on the third day post-infection were cultured on Sabouraud agar medium to assess fungal loads, the corneal homogenates from the DMSO and 25 μM isoimperatorin intervention group cultured a large number of A. fumigatus colonies (Supplementary Figure 4).

The above experiments demonstrate that there is no clear evidence to suggest that 25 μM isoimperatorin has antifungal activity.

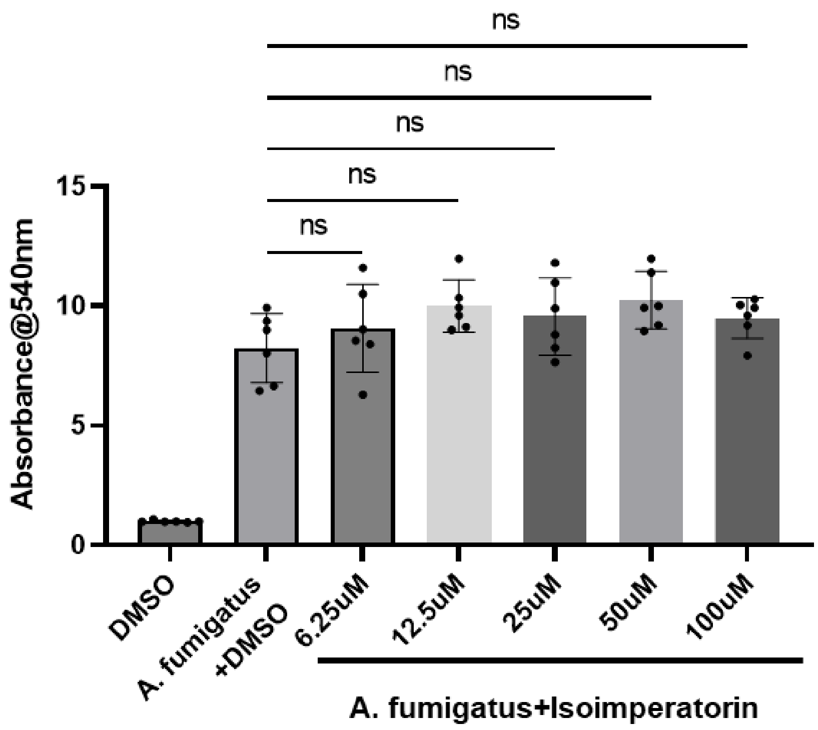

Supplementary Figure 1. Anti-A. fumigatus action of isoimperatorin.

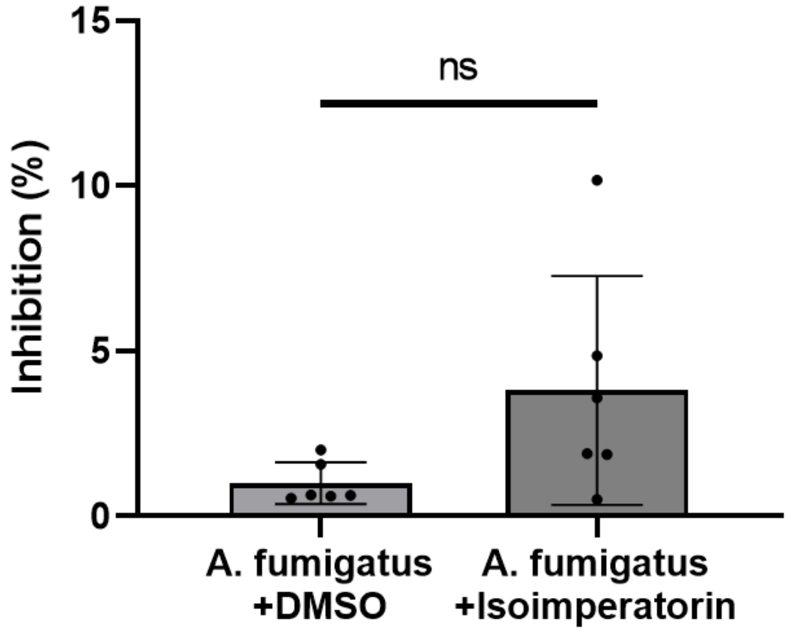

Supplementary Figure 2. Influence of 25 μM isoimperatorin on inhibiting the growth of A. fumigatus biofilm.

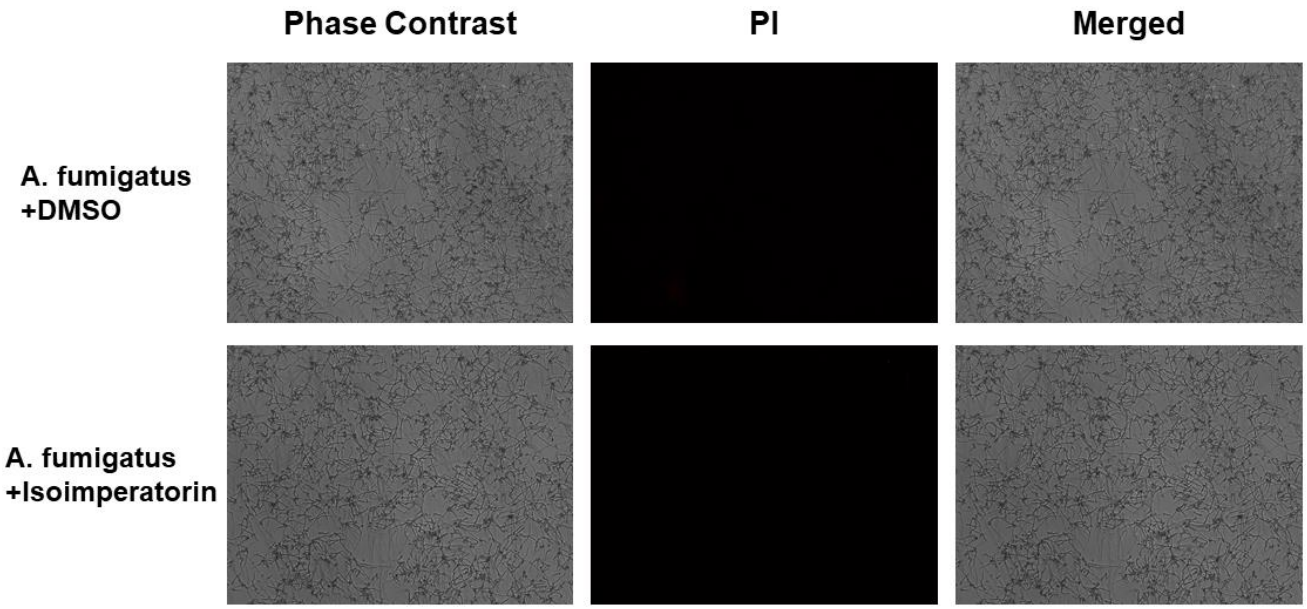

Supplementary Figure 3. Impact of 25 μM isoimperatorin on the fungal cell membrane.

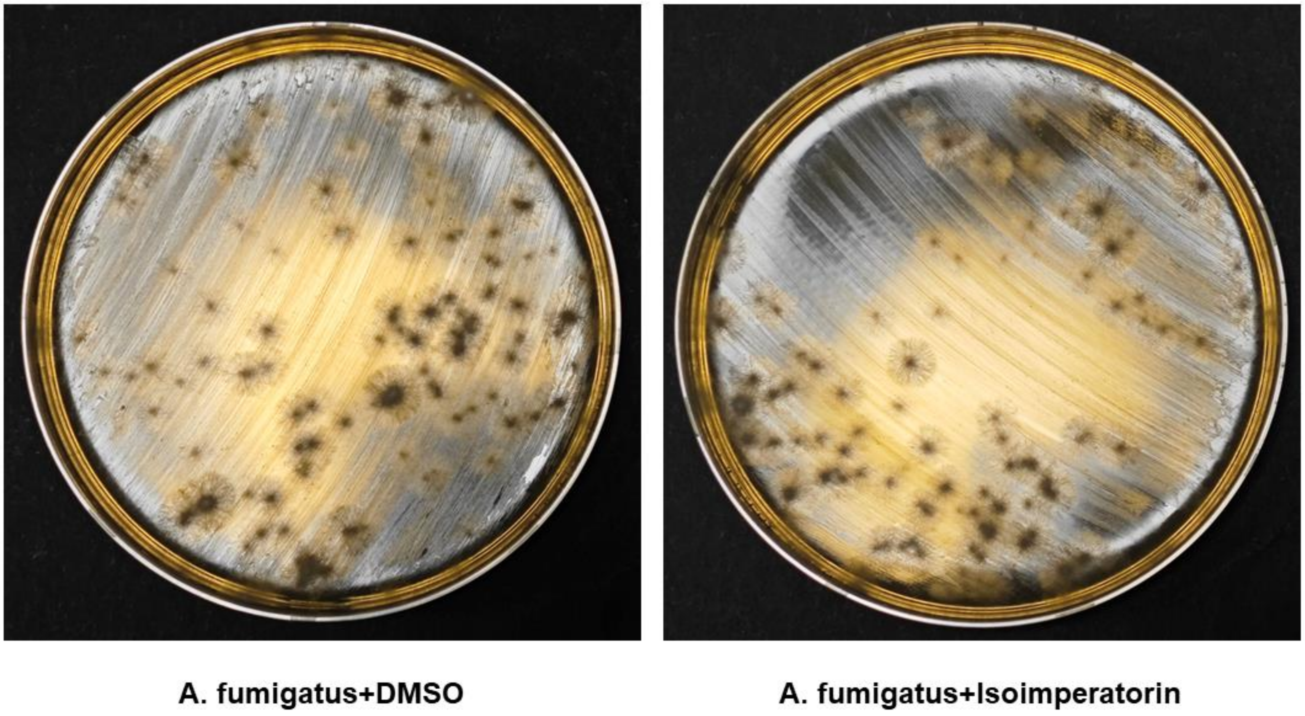

Supplementary Figure 4. Plates of viable fungus in the cornea 3 days after infection.

2 Supplementary Tables

	Grade Ⅰ	Grade Ⅱ	Grade Ⅲ	Grade Ⅳ
**Ulcer area**	1-25%	26-50%	51-75%	76-100%
**Turbidity**	The iris texture is still clear, and the pupil shows mild turbidity	Visible iris, relatively cloudy cornea	Iris is invisible, cornea is unevenly cloudy	Uniform corneal opacity
**Ulcer morphology**	Mild irregularity	Mild edema, irregular	Severe edema, niche like ulcers or bulging of the posterior elastic layer	Perforation or bulging of the posterior elastic layer
**Rating**	1	2	3	4

Supplementary Table 1. Grading of Inflammation Degree in Mouse Fungal Keratitis Model.

Sequences of primers used are provided.

Gene	Primer sequence (5′ – 3′)
mβ-actin-F	GAT TAC TGC TCT GGC TCC TAG C
mβ-actin-R	GAC TCA TCG TAC TCC TGC TTG C
mTNF-α-F	ACC CTC ACA CTC AGA TCA TCT T
mTNF-α-R	GGT TGT CTT TGA GAT CCA TGC
mNOS2-F	TGG ACC TCA AGA TGT CCA CA
mNOS2-R	GGT CTC CAG AGG TTC TTC CA
mARG-1-F	TGG GTG ACT CCC TGC ATA TCT
mARG-1-R	TTC CAT CAC CTT GCC AAT CC
mYM-1-F	GGG CAT ACC TTT ATC CTG AG
mYM-1-R	CCA CTG AAG TCA TCC ATG TC
mFIZZ-1-F	TCC CAG TGA ATA CTG ATG AG
mFIZZ-1-R	CCA CTC TGG ATC TCC CAA GA

Supplementary Table 2. Nucleotide Sequences of Mouse Primers for qRT-PCR.
